# Integrative analysis of single-cell and bulk RNA seq to reveal the prognostic model and tumor microenvironment remodeling mechanisms of cuproptosis-related genes in colorectal cancer

**DOI:** 10.18632/aging.205324

**Published:** 2023-12-08

**Authors:** Bowen Chu, Yaohui Wang, Jiwen Yang, Bohan Dong

**Affiliations:** 1 Clinical School, Wannan Medical College, Wuhu 241000, Anhui, P.R. China; 2 Department of Microbiology and Immunology, Wannan Medical College, Wuhu 241000, Anhui, P.R. China; 3 Department of Nuclear Medicine, Yijishan Hospital of Wannan Medical College, Wuhu 241000, Anhui, P.R. China

**Keywords:** colorectal cancer, cuproptosis, copper metabolism, tumor microenvironment, t cell exhaustion

## Abstract

Background: Recently, there has been a great deal interest in cuproptosis, a form of programmed cell death that is mediated by copper. The specific mechanism through which cuproptosis-related genes impact the development of colorectal cancer (CRC) remains unknown.

Methods: Here, we combined bulk RNA-seq with scRNA-seq to investigate the CRGs functions within CRC. A number of 61 cuproptosis-related genes were chosen for further investigation. Nine prognostic CRGs were identified by Lasso-Cox. The RiskScore was created and the patients have been separated into two different groups, low- and high-RiskScore group. The CIBERSORT, ESTIMATE, MCP-counter, TIDE, and IPS have been employed to score the TME, and GSVA and GSEA were utilized to evaluate the pathway within the both groups. Further, we used cell communication analysis to explore the tumor microenvironment remodeling mechanisms of the COX17 and DLAT based on scRNA-seq. Finally, we used IHC and qPCR to validate the expression of COX17 and DLAT.

Results: AOC3, CCS, CDKN2A, COX11, COX17, COX19, DLD, DLAT, and PDHB have been recognized as prognostic CRGs in CRC. The high-risk group exhibited the worst prognosis, an immune-deficient phenotype, and were more resistant to ICB treatment. Further, scRNA-seq analysis revealed that elevated expression of COX17 in CD4-CXCL13Tfh could contribute to the immune evasion while DLAT had the opposite effect, reversing T cell exhaustion and inducing pyroptosis to boost CD8-GZMKT infiltration.

Conclusions: The current investigation has developed a prognostic framework utilizing cuproptosis-related genes that is highly effective in predicting prognosis, TME type, and response to immunotherapy in CRC patients. Furthermore, our study reveals a novel finding that elevated levels of COX17 expression within CD4-CXCL13 T cells in CRC mediates T cell exhaustion and Treg infiltration, while DLAT has been found to facilitate the anti-tumor immunity activation through the T cell exhaustion reversal and the induction of pyroptosis.

## INTRODUCTION

Globally, colorectal cancer has emerged as the second most lethal form of cancer. The prevalence of colon cancer is rising, and the tendency toward occurrence in the young is becoming more and more pronounced, as a result of changes in people’s lifestyles and higher living standards [1]. In addition to this, the prognosis for colorectal cancer patients is very grim. the 5-year survival rate for Stage IV colorectal cancer patients is only 11%-15% [2, 3]. However, effective therapies for CRC are still limited to colectomy, radiotherapy and adjuvant chemotherapy. These therapies have very limited effects and are associated with more serious side effects, such as neuropathy and chemotherapy-related diarrhea, especially oxaliplatin [3, 4]. In recent years, immunotherapy, especially anti-PD-1/PD-L1, has been prominent in the treatment of high-frequency MSI/ mismatch repair-deficient (MSI-H/dMMR) CRC patients [5]. However, this therapy is ineffective in a significant proportion of patients, most likely due to the complex tumor microenvironment (TME) of colorectal cancer patients. It is generally known that clinical prognosis, anti-tumor immunotherapy, and tumor-infiltrating lymphocytes (TILs) infiltration in tumor tissue are all closely associated [6, 7]. While TIL infiltration is lower in the tissues of cancer patients with microsatellite stability (MSS), resulting in low responsiveness to antitumor immunotherapy, within the context of colorectal cancers that exhibit MSI are characterized by the accumulation of somatic mutations throughout the DNA, rendering them more susceptible to infiltration by TILs [8, 9]. Thus, the investigation of new immune-related indicators is imperative for the purpose of prognosticating clinical findings and determining the immunotherapy effectiveness in cases of colorectal cancer. The effectiveness of tumor immunotherapy has been demonstrated to be enhanced by ferroptosis-related metabolism in the past [10], but the role of copper metabolism in immunotherapy has not been explored. Due to its redox characteristics, copper, a vital nutrient, may be both helpful and harmful to cells [11]. It has been demonstrated that compared to healthy tissues, tumor tissues require higher concentrations of copper to promote cell proliferation and metastasis [12]. According to a recent study, the promotion of a unique model of regulated cell death (RCD) called cuproptosis is facilitated by intracellular copper, and it is different from the more conventional models of cell death include apoptosis, ferroptosis, pyroptosis, as well as necrosis [13]. The direct interaction between copper and the lipidated constituents of the tricarboxylic acid (TCA) cycle is has performed a crucial role within the cuproptosis process. This allows the aggregation of lipidated proteins and consequent depletion of proteins containing iron-sulfur clusters, hence, the induction of proteotoxic stress leads to cellular dysfunction and ultimately leads to apoptotic or necrotic cell death [13]. More specifically, under the regulation of the FDX1, LIAS linked lipoyl moiety to DLAT. When excess copper ions enter the cell, these copper ions bind to lipoyl moiety of lipoylated DLAT to oligomerize DLAT and further proteotoxic stress and eventually cell death [14]. The cuproptosis-related genes (CRGs) effect on the growth, proliferation, and prognostication of colorectal cancer remains unclear. It is also necessary to investigate their mechanisms as possible antitumor immunotherapy targets.

Apart from the cancer cells malignant proliferation, TME comprises diverse immune cells, which are essential for the tumor cells development as part of the surrounding microenvironment. In the context of tumor immunity, tumor cell-mediated cellular immunity performs a vital function which can be categorized into various subtypes such as CD4+ T cells, CD8+ T cells, Treg cells, etc. [15]. CD4+ T cells, which act as helper cells, possess the capacity to enhance the anti-tumor immune response of CD8+ T cells. However, Treg cells possess the capacity to suppress the anti-tumor immune response and are often highly correlated with clinical patient prognosis and treatment outcome [16]. Recent investigations have demonstrated the significant involvement of copper ions in both humoral and cellular immunity [17]. However, the precise process through which CRGs affect immune cells in the tumor microenvironment remains to be clarified.

Throughout this research, the correlation among cuproptosis-related genes and the colorectal cancer prognosis was investigated through an analysis of public databases, for this reason, a prognostic model for colorectal cancer was developed utilizing cuproptosis-related genes, and further elucidated the specific mechanisms by which the risk gene COX17 and the protective gene DLAT affect immune cells through the level of a single-cell to improve the therapeutic efficacy and prognosis for colorectal cancer.

## RESULTS

### Study of cuproptosis related genes in CRC

**Figure 1:**
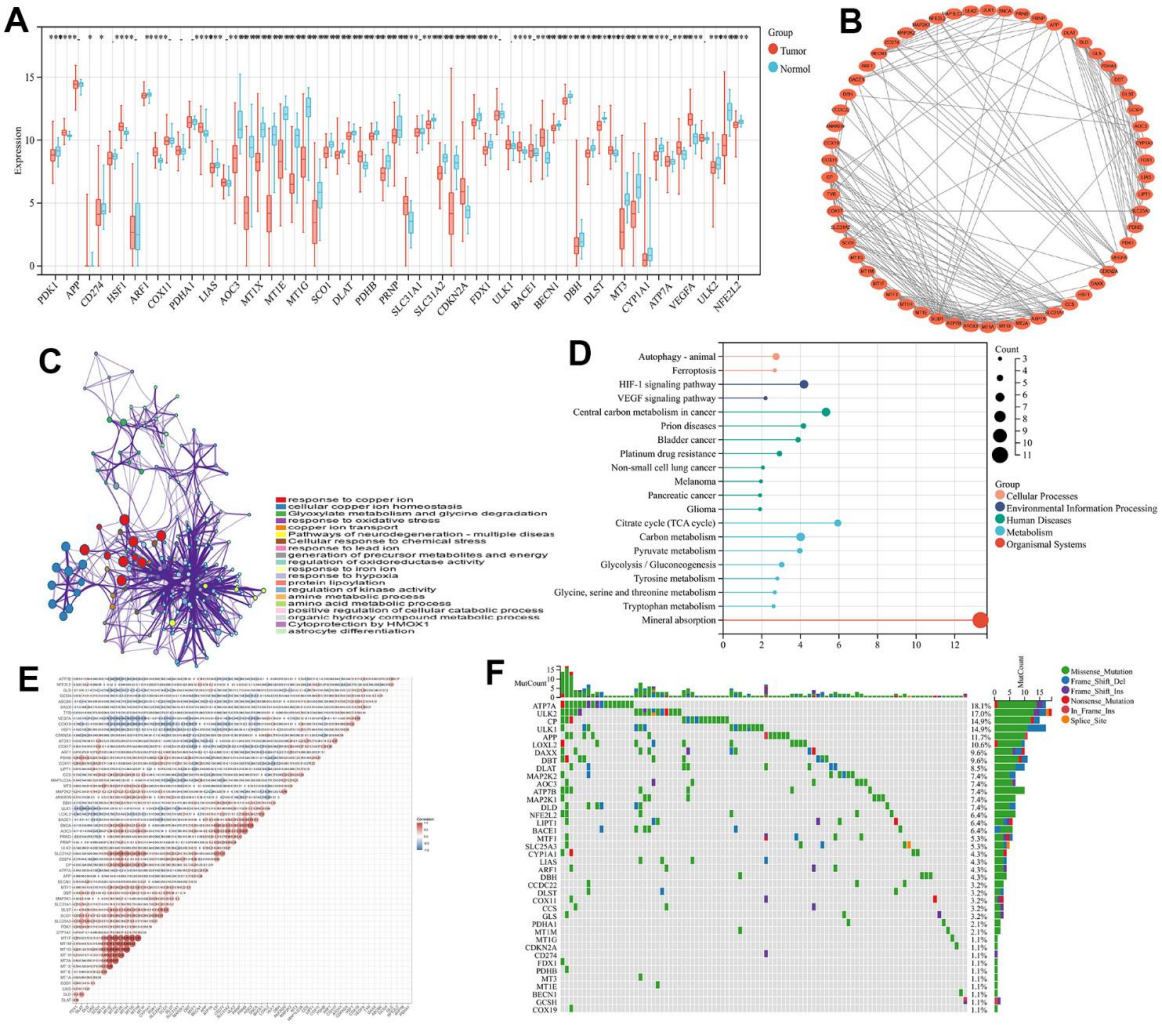
**Study of the cuproptosis-related genes within CRC.** (**A**) 61 CRGs transcriptional difference among normal and tumor tissues (**B**) Interactions among CRGs. The lines among the genes indicate their interactions. (**C**) GO analyses of CRGs (**D**) KEGG analyses of CRGs (**E**) Correlation analysis of CRGs (**F**) The waterfall plot of tumor somatic mutations of CRGs.

For examining 61CRGs expression levels in cases of colorectal cancer, at first, we started by retrieving information on expression profiles from the TCGA database. Upon conducting a comparative analysis of the 61 cuproptosis-related genes expression levels within the patient’s tumor tissues and normal tissues, it was observed that 31 genes exhibited differential expression. The expression of COX11, COX17, LIAS, CDKN2A and AOC3 was significantly higher in tumor tissues than in normal tissues. However, DLAT, PDHB, DLST, FDX1, SLC31A2, DLD and DBH were more highly expressed in normal tissues. (Figure 1A, and Supplementary Table 1). The STRING database was utilized to investigate the interaction among these gene-encoded proteins, using the Cytoscape 3.9.1 software, the PPI network was mapped. The findings indicate that the 61 CRGs exhibited strong connectivity (Figure 1B, and Supplementary Figure 1). Also, in order to investigating the cuproptosis-related genes biological functions, we performed gene set enrichment analyses using GO and KEGG databases. Response to copper ion, cellular copper in homeostasis, response to oxidative stress, copper ion transport signaling pathways in GO are enriched. Mineral absorption, citrate cycle (TCA cycle), central carbon metabolism in cancer, HIF-1 signaling pathway signaling pathways in KEGG are enriched. (Figure 1C–1E and Supplementary Table 2) These metabolic and transport routes for copper are linked to mitochondrial metabolism, tumor growth, and metastasis. We further investigated the copy number variants (CNV) and somatic mutations in CRGs in cases of colorectal cancer. The result found that 132 of 399 samples (33.1%) showed genetic variation. ATP7A was the predominantly mutated gene with the 18.1% mutation frequency (Figure 1F). All of these results suggested that the potential role of CRGs in colorectal cancer deserves to be explored in depth.

### Construction of the CRGs-related prognostic model

**Figure 2:**
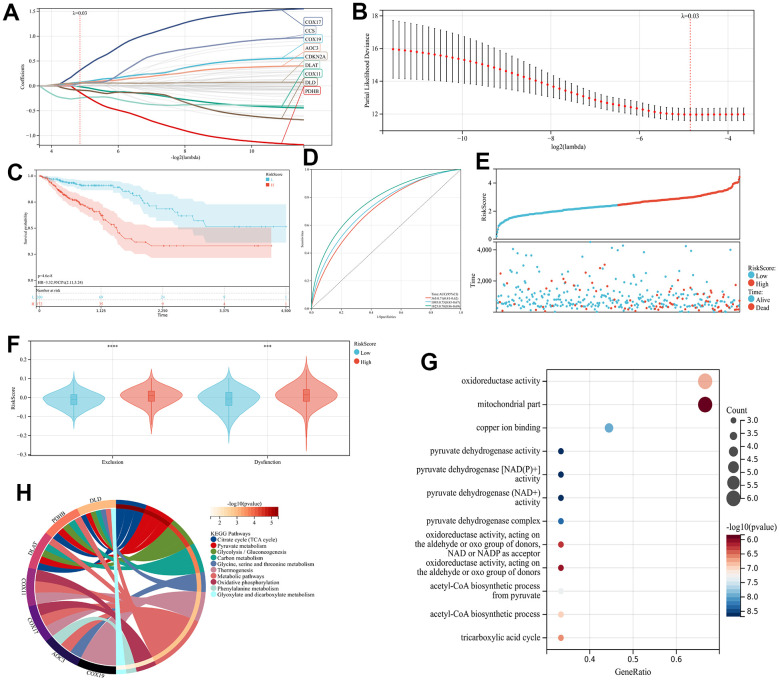
The CRGs-related prognostic Model construction (**A**, **B**) The LASSO regression analysis and partial likelihood deviance on the CRGs (**C**) KM and (**D**) ROC curves demonstrating the predictive significance within the training cohort. (**E**) The RiskScore and survival result of each case (**F**) TIDE scores of high- and low-risk groups (**G**) KEGG analyses of 9 prognostic CRGs (**H**) GO analyses of 9 prognostic CRGs.

Sixty-one genes were screened for significant prognostic relevance using LASSO analysis to construct a prognostic model (Figure 2A, 2B, p<0.05), and finally AOC3, CCS, CDKN2A, COX11, COX17, COX19, DLD, DLAT, and PDHB were recognized as signature genes. Enrichment analysis was conducted on the nine genes using GO and KEGG methods, where GO was primarily furnished with parts connected to mitochondrial and oxidative reactions, such as oxidoreductase activity and mitochondrial parts (Figure 2G). The majority of the signaling pathways associated with substance metabolism found in KEGG include glycolysis/gluconeogenesis, pyruvate metabolism, carbon metabolism, the citrate cycle (TCA cycle), and oxidative phosphorylation. (Figure 2H). The nine signature genes expression levels and their corresponding coefficient values were utilized in the calculation of risk scores, in accordance with the subsequent formula: RiskScore=∑i=1 nCoef(Xi)×Exp(Xi). The individuals diagnosed with CRC have been grouped into high and low-risk groups relying on their RiskScores as presented in (Supplementary Table 3), and the Kaplan-Meier curves exhibited a statistically significant variation in prognosis between patients categorized as high-risk and those categorized as low-risk, with those with higher risk exhibiting a poorer outcome (Figure 2C, p<0.001), while a higher frequency of mortality events was observed throughout the high-risk group (Figure 2E). The prognostic model’s predictive capacity for the prognosis of patients with colorectal cancer was evaluated using Receiver Operating Characteristic (ROC) curves. The results showed that the Area Under the Curve (AUC) values for 1-year, 3-year, and 5-year survival were 0.71, 0.75, and 0.78, respectively (Figure 2D). Based on further TIDE algorithm assessment of immunotherapy responsiveness in the two groups, individuals categorized as high-risk patients exhibited a greater tendency to develop resistance to immunotherapy (Figure 2F, p<0.001).

### Validation of prognostic models

**Figure 3:**
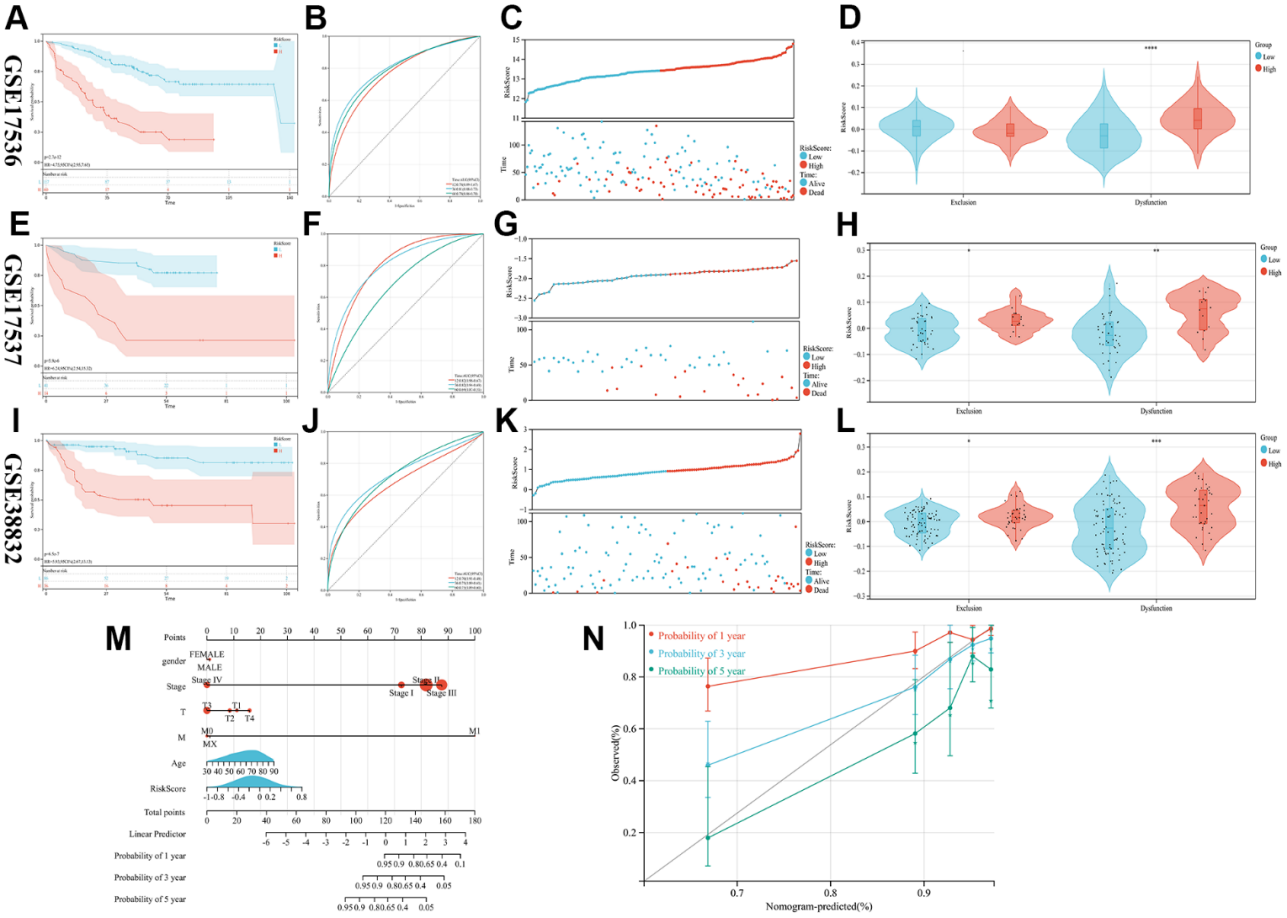
**Validation of the prognostic model.** (**A**–**L**) The KM, ROC, RISKSCORE, and TIDE analysis showing the prognostic value in three cohorts (**M**) Construction of the nomogram model utilizing RiskScore and other characteristics. (**N**) A calibration plot compares nomogram-predicted survival rates with observed survival rates.

Three GEO cohorts (GSE17536, GSE17537, and GSE38832) were used as validation sets to calculate the RiskScore according to the previous formula. CRC patients in these cohorts were categorized into groups based on their level of risk, with some being categorized as high-risk while others are categorized as low-risk. A comparison was made between the two groups in terms of to the probability of survival, survival status, and response to immunotherapy. The study reveals that the high-risk group exhibited a poorer prognosis through all three validation cohorts (Figure 3A, 3C, 3E, 3G, 3I, 3K), and the predictive model prognostic value was found to be significant for both short-term and long-term follow-up periods by plotting time-dependent ROC curves and calculating AUC at different time points (Figures 3B, 3F, 3J). Finally, in order to assess the individual risk of patients with TCGA-CRC, a personalized scoring chart was developed for predicting overall survival using four distinct parameters: gender, age, tumor stage, and risk score (Figure 3M). In addition, the calibration plot revealed that the nomogram conformed to the ideal model (Figure 3N).

### Immune activity comparison between subgroups

**Figure 4:**
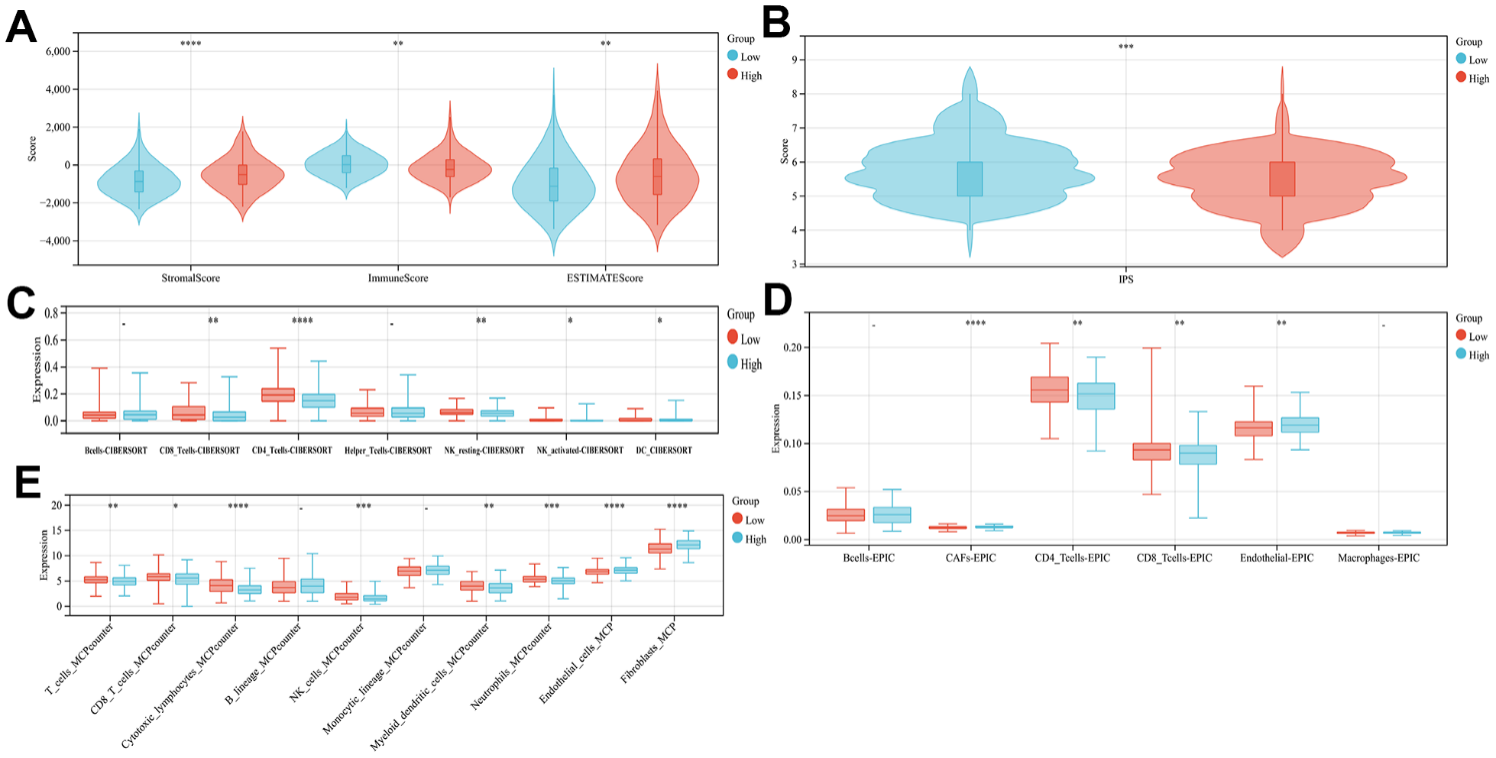
**Comparison of the subgroup's immune activity.** (**A**) The comparison of stromal, immune, as well as estimate scores among the low- and high-risk score groups (**B**) Comparison of the IPS score in high- and low-risk groups. Immuno-infiltration analysis using CIBERSORT (**C**), EPIC (**D**), and MCPcounter (**E**).

In order to examine the variations in immune infiltration within the tumor microenvironment among the high- and low-risk groups, the application of ESTIMATE analysis revealed reduced stromal scores (p<0.001) and higher immune scores (p<0.001) in the low-risk group, indicating the immune cell infiltration elevated levels through the low-risk group (Figure 4A). Three immune infiltration analyses, CIBERSORT, EPIC, and MCP-counter, have been conducted on the TCGA cohort, revealing a significant variance in immune status between both, the high-risk group and the low-risk group. The low-risk patient group exhibited a higher prevalence of CD8+ T cells, CD4+ T cells, neutrophils, NK and NKT cells infiltration, whereas the group of individuals which categorized as high-risk showed a greater prevalence of fibroblast, endothelial cells, and fibroblasts (CAF) cells infiltration (Figure 4C–4E, and Supplementary Figure 2 p<0.05). The tumor may be suppressed through cellular immune mechanisms involving CD4+ T cells, CD8+ T cells, and NK cells, while CAF, as a major part of the tumor stroma, played a crucial function in tumor immune evasion [18]. Suggesting that our high-risk group may be correlated with the development of an immunosuppressive microenvironment. Finally, upon comparing the two groups IPS scores, it was observed that the low-risk individuals exhibited significantly greater scores (Figure 4B, p<0.01), Indicating that individuals with a lower risk profile may exhibit a more favorable reaction to immune checkpoint blockade therapy.

### Analysis of RiskScore-related signal pathways

**Figure 5:**
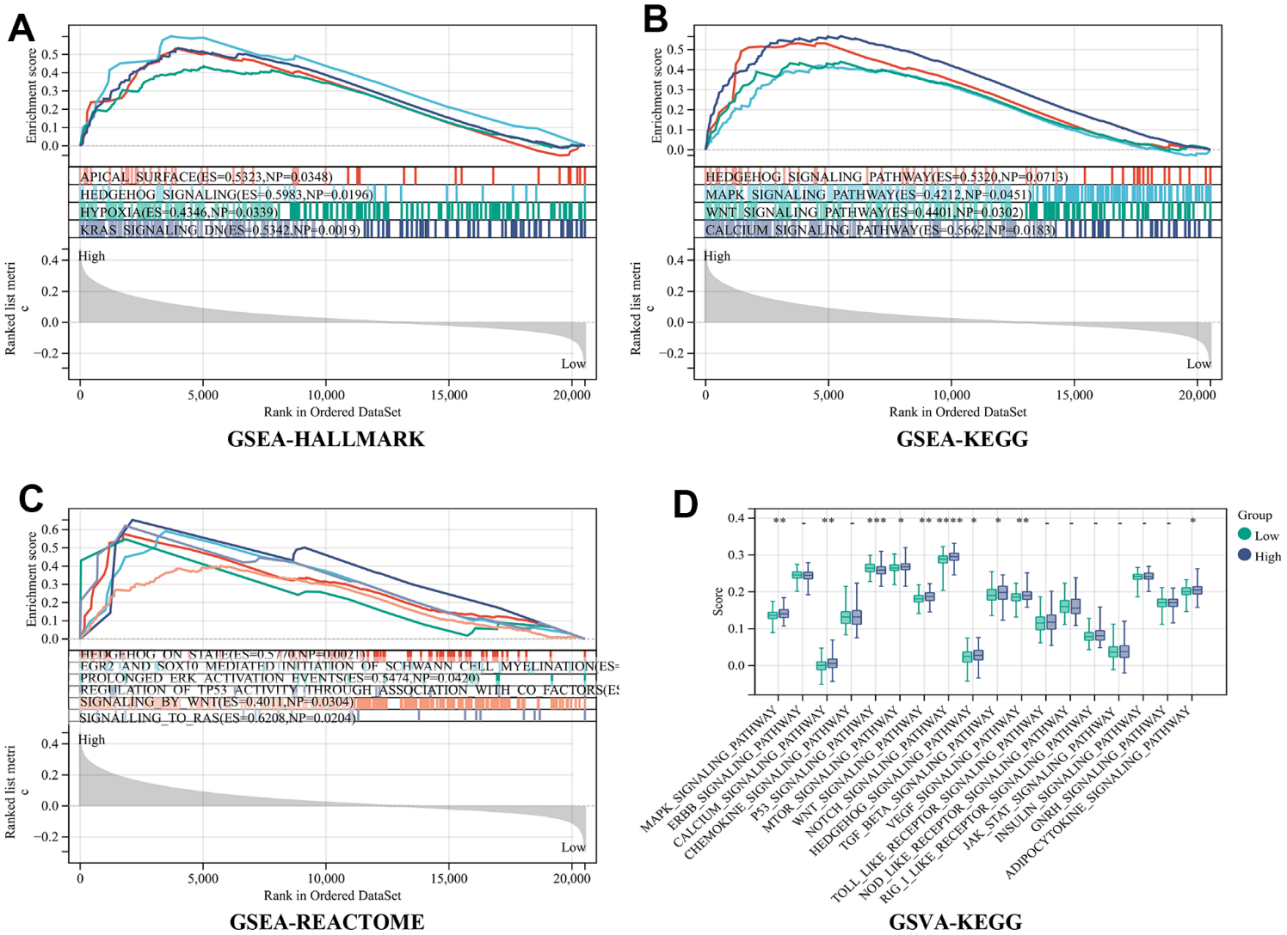
**Analysis of RiskScore-related signal pathways.** (**A**) Hallmark (**B**) KEGG (**C**) REACTOME enrichment analysis by gene set enrichment analysis (GSEA) (**D**) KEGG enrichment analysis by the Gene Set Variation Analysis (GSVA).

The groups categorized as high-risk and low-risk were subjected to gene set enrichment analyses utilizing GSEA and GSVA analyses. The results obtained from GSEA revealed that in high-risk group APICAL SURFACE, HYPOXIA, HEDGEHOG SIGNALING, and KARAS SIGNALING were activated in the hallmark gene set (Figure 5A). WNT SIGNALING PATHWAY, HEDGEHOG SIGNALING PATHWAY, and CALCIUM SIGNALING PATHWAY were activated in the KEGG gene set (Figure 5B); HEDGEHOG ON STATE, EGR2 AND SOX10 MEDIATED INITIATION OF SCHWANN CELL MYELINATION, PEGULATION OF TP53 ACTIVITY THROUGH ASSOCIATION WITH CO FACTORS, SIGNALING BY WNT and SIGNALLING_TO_RAS were upgraded in the REACTOME gene set (Figure 5C). The outcomes obtained from GSVA showed that MAPK_SIGNALING_PATHWAY, ERBB_SIGNALING_PATHWAY, MTOR_SIGNALING_PATHWAY, WNT_SIGNALING_PATHWAY, NOTCH_SIGNALINGPATHWAY, HEDGEHOG_SIGNALING_PATHWAY, TGF_BETA_SIGNALING_PATHWAY, VEGF_SIGNALING_PATHWAY were observed to be active within the high-risk group (Figure 5D). Among them, numerous pathways associated with progression and metastasis of cancer were determined, that have been significantly activated within the high-risk group, including the WNT pathway connected with tumor invasion and proliferation and the HEDGEHOG pathway involved in tumor angiogenesis.

### Survival analysis of 9 CRGs

We further validated the relationship between the nine genes selected by lasso-cox using the KM plot. All of them exhibited a significant association with the prognosis of individuals diagnosed with CRC (p<0.05), where patients in the high expression groups of AOC3 (Supplementary Figure 3A), CCS (Supplementary Figure 3B), CDKN2A (Supplementary Figure 3C), COX17 (Supplementary Figure 3E), and COX19 (Supplementary Figure 3F) exhibited unfavorable prognosis. Individuals in the high expression groups of COX11 (Supplementary Figure 3D), DLAT (Supplementary Figure 3G), DLD (Supplementary Figure 3H), and PDHB (Supplementary Figure 3I) had a better prognosis.

### Genetic features of RiskScore and tumor somatic mutation of CRC

**Figure 6:**
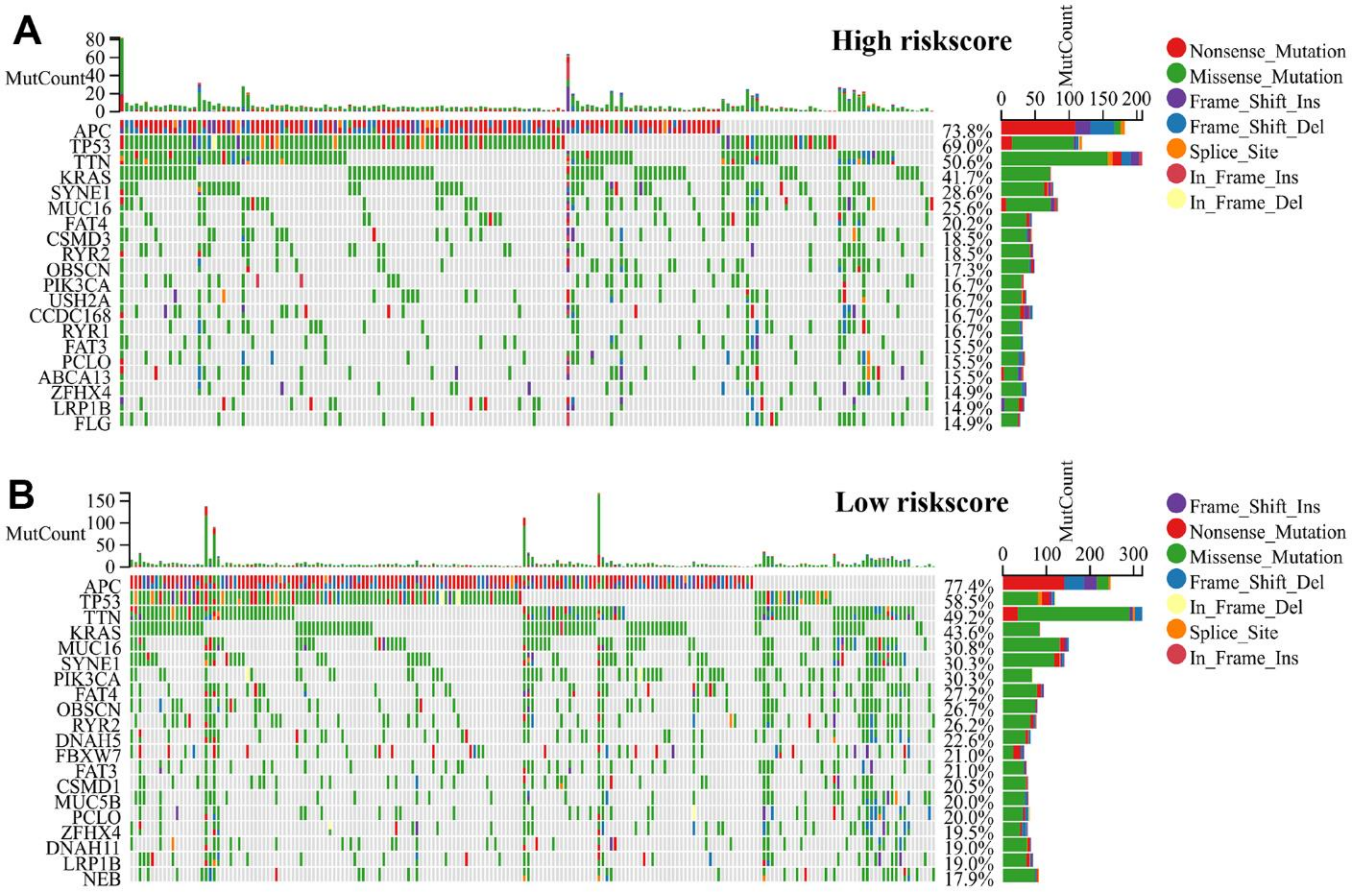
**RiskScore genetic features and somatic mutation of the CRC.** (**A**) Tumor somatic mutation within the high-risk group and (**B**) the low-risk group revealed by the waterfall.

Tumors with low somatic mutation rates tend to have low immunogenicity, inducing low anti-tumor immune responses in the body and making immunotherapeutic regimens against that tumor insensitive. Based on the somatic mutation waterfall plots within the high-risk group and low-risk group, our findings indicate that the individuals categorized in the low-risk group exhibited a greater incidence to the mutations number in comparison to those related to the high-risk group. (Figure 6A, 6B).

### Expression and subcellular localization of COX17 and DLAT

**Figure 7:**
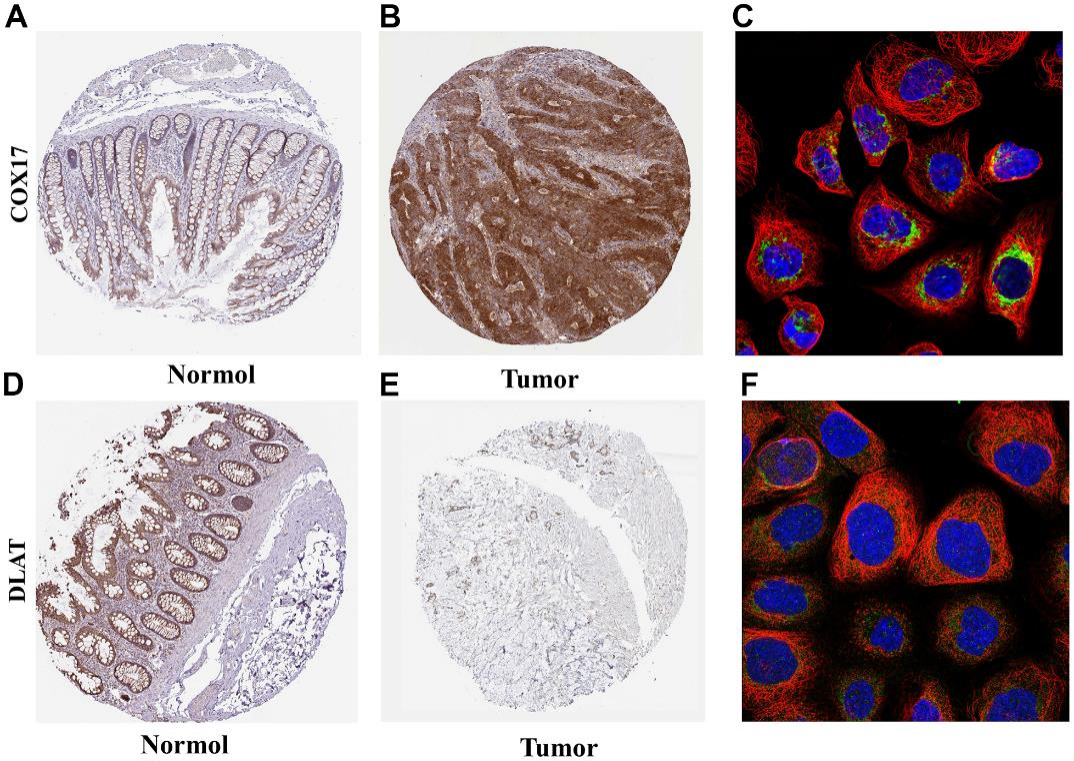
Expression and subcellular localization of COX17 and DLAT (**A**) The expression of COX17 is greater in tumor tissues compared to (**B**) normal tissues. (**C**) COX17 was mainly located in mitochondria. DLAT expression in (**D**) CRC and (**E**) normal issues. (**F**) DLAT were mainly located in the mitochondria by the HPA database analysis.

Next, the two hub genes with the highest |Coef| values, namely risk factor COX17 and protective factor DLAT, were screened based on the Lasso analysis for further analysis. (Supplementary Table 4) First, the IHC results showed higher expression of COX17 (Figure 7A, 7B) and lower expression of DLAT within tumor tissues than normal tissues. The qPCR experiments were also carried out to detect the expression levels of these two genes in HCT116 and NCM-460 cells, and the results were consistent with previous IHC results (Supplementary Figure 4, p<0.05). Furthermore, the subcellular localization maps of COX17 (Figure 7C) and DLAT (Figure 7F) showed that both genes were mainly localized in mitochondria, suggesting that the occurrence of cuproptosis was closely related to mitochondrial metabolism.

### Pan-cancer survival and functional analysis of COX17 and DLAT

**Figure 8:**
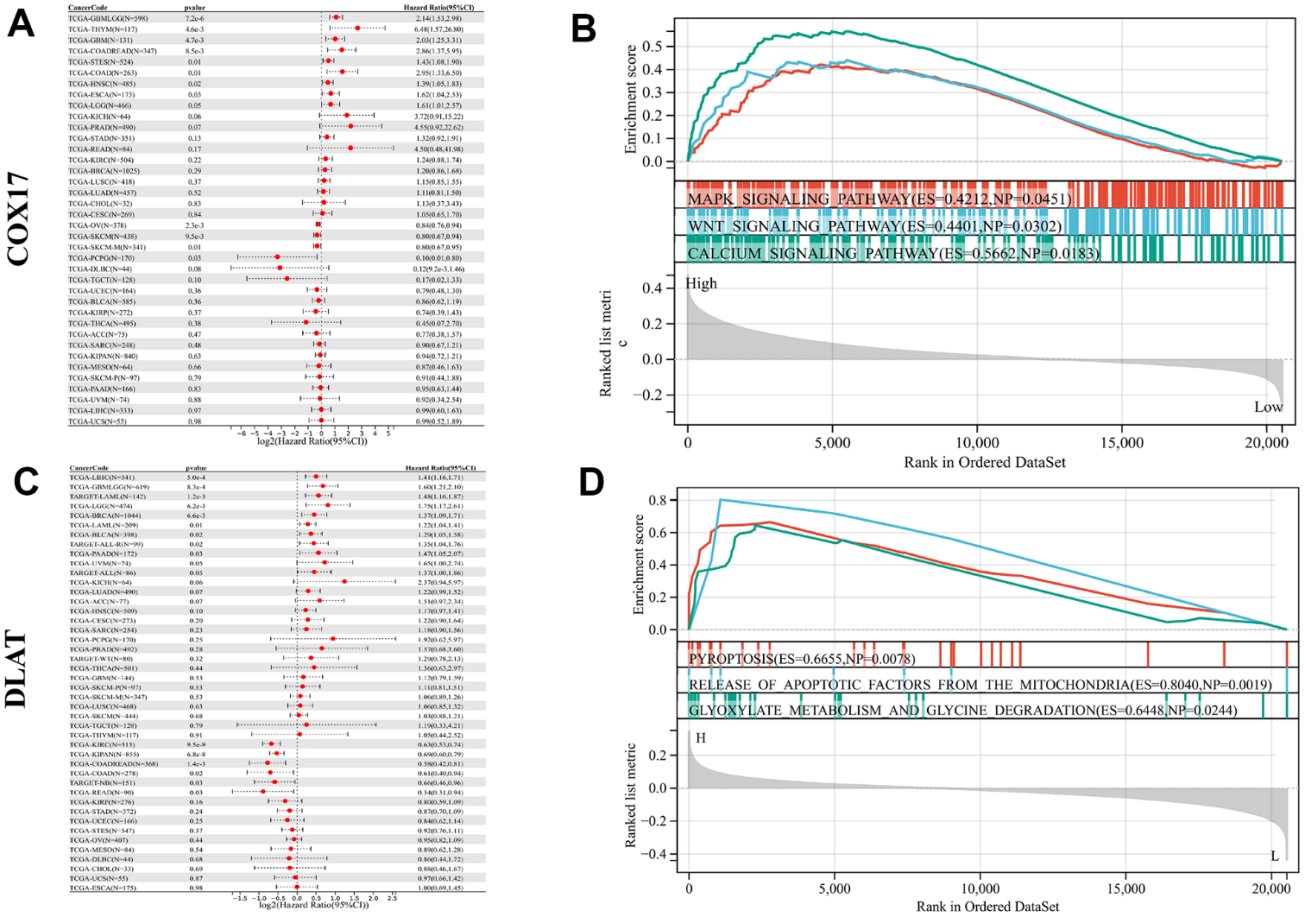
**Pan-cancer survival and functional analysis of COX17 and DLAT.** A forest plot shows the risk degree of COX17 (**A**) by the uni-Cox. Genomic enrichment analysis (GSEA)-based KEGG functional enrichment for the expression of COX17 (**B**) in CRC. A forest plot shows the risk degree of DLAT (**C**) by the uni-Cox. GSEA-based KEGG functional enrichment for DLAT (**D**) in CRC.

The relationship between COX17 and DLAT and overall survival (OS) in 32 cancer patients was analyzed and found that COX17 and DLAT expression were significantly associated with OS in many cancers. Among them, COX17 was identified as a potential risk factor in GBM(p<0.001) and CRC(p<0.001), however, in other types of cancer, it possesses a protective function, especially PCPG (p<0.05) (Figure 8A). DLAT exhibited a protective effect in CRC(p<0.001), but in LIHC (p<0.001), GBMLGG (p<0.001), LGG (p <0.001) and BRCA (p<0.001) (Figure 8C) was a risk factor suggesting that there is often heterogeneity among our tumors. It is especially important to find tumor treatment-specific targets. Additionally, for evaluating the enrichment of pathways in groups with high and low expression of COX17 and DLAT, the GSEA was employed, and the outcomes showed that MAPK_SIGNALING_PATHWAY (p<0.05) and WNT_SIGNALING_PATHWAY (p<0.05) were significantly activated in the COX17 high expression group (Figure 8B). Within the context of the DLAT high expression group, PYROPTOSIS (p<0.01) and RELEASE OF APOPTOTIC FACTORS FROM MITOCHONDRIA were upregulated (Figure 8D). Surprisingly, DLAT actually linked pyroptosis to cuproptosis, by inducing mitochondrial apoptosis and further active Apaf-1-caspase-3-GSDME pathway (Supplementary Figure 5, p<0.05) through which mitochondrial damage induce pyroptosis [19]. Some studies have revealed that a minor proportion of cancer cells experiencing pyroptosis can adequately modulate the tumor immune microenvironment, thereby inducing a potent T-cell-mediated antitumor immune response [20]. This suggests that DLAT may shaping an immunoactive TME.

### Single-cell analysis of COX17 and DLAT expression in the immune microenvironment of colorectal carcinoma

**Figure 9:**
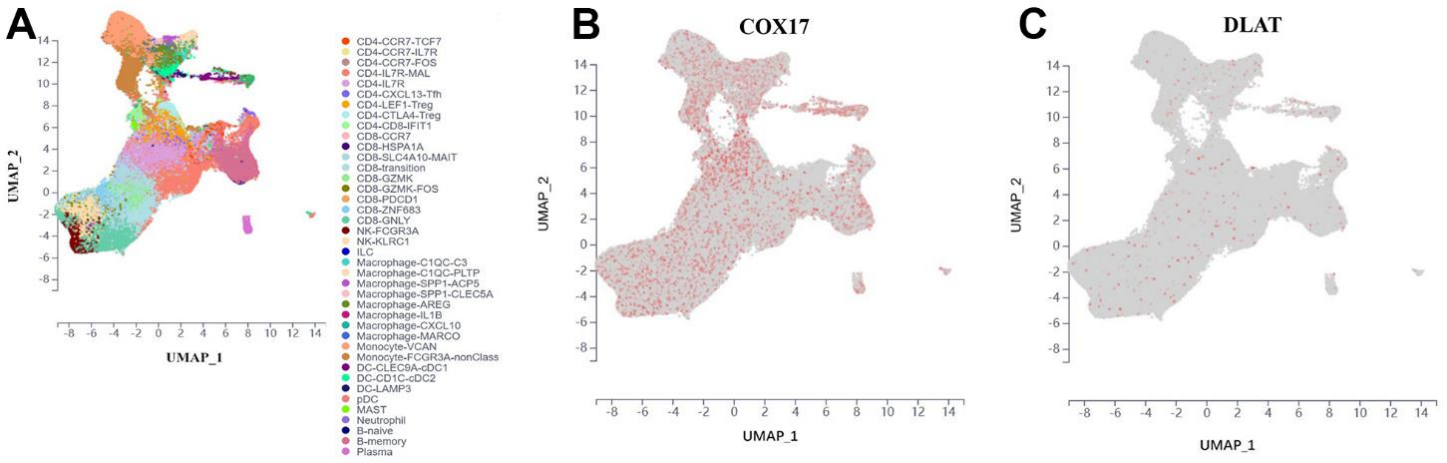
**Introduce the outcomes of a single-cell analysis carried out to examine COX17 and DLAT expression levels within the immune microenvironment of colorectal cancer.** (**A**) UMAP plot illustrates the 40 cell types distribution and dissimilarity. (**B**) COX17 is highly expressed in TME. (**C**) DLAT is expressed in small amounts in each cell type.

For further discover the function of COX17 and DLAT on TME, we chose a scRNA-seq data set which was collected from tumor tissue and peripheral blood samples of 14 treatment-naïve CRC patients, containing 200,626 cells totally. We classified cells from CRC patients into 40 clusters, such as CD4+ T cells, CD8+ T cells, lymphocytes, phagocytes, monocytes, dendritic cells (DC), mast cells, natural killer (NK) cells, neutrophils, plasma cells, and B cells (Figure 9A), which demonstrated that COX17 presented a significant degree of expression in the tumor microenvironment while DLAT expression levels were low (Figure 9B, 9C). We further analyzed the expression of two genes in different cells. The results showed that COX17 was highly expressed in malignant cell, epithelial cell, fibroblast, neutrophil, CD4-CXCL13-T cell, CD4-CTLA4-Treg, CD8-PDCD1-T cell (Supplementary Figure 6). However, the overall expression of DLAT was low in TME and was only expressed in cells such as malignant cells, CD4-CCR7-T cells and CD8-CCR7-T cells (Supplementary Figure 7).

### CD4-CXCL13-Tfh drives suppressive tumor microenvironment formation through upregulating the expression of COX17

**Figure 10:**
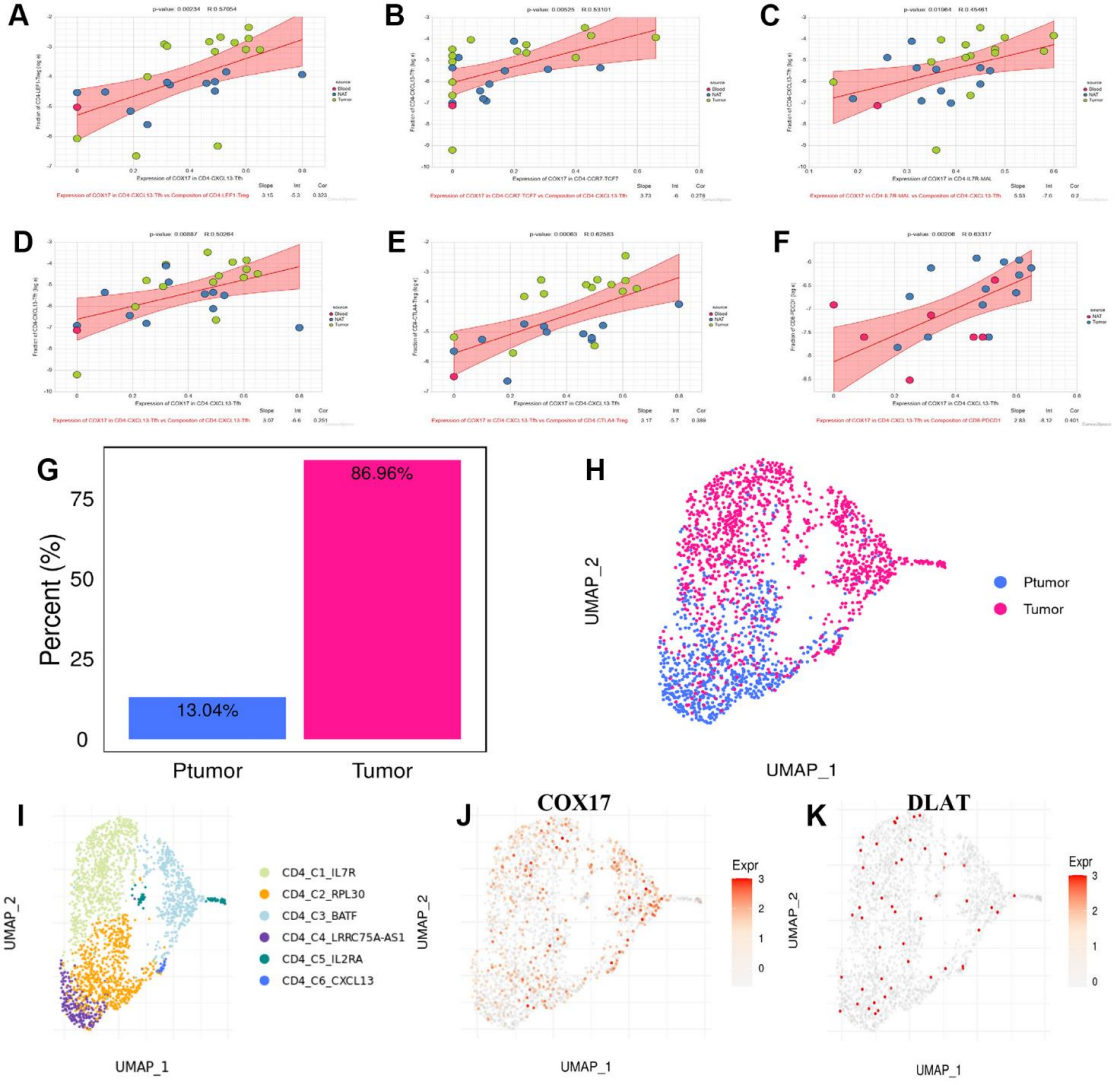
CD4-CXCL13-Tfh drives suppressive tumor microenvironment formation through upregulating the COX17 expression (**A**–**F**) Association between expression of COX17 and cell composition (**G**) Histogram of CD4-CXCL13-T composition in cancer and paracancer (**H**) UMAP map of cancer and paracancer (**I**) UMAP show the landscape of cell types of CD4^+^T clusters. (**J**) Expression and distribution of COX17 and (**K**) DLAT.

**Figure 11:**
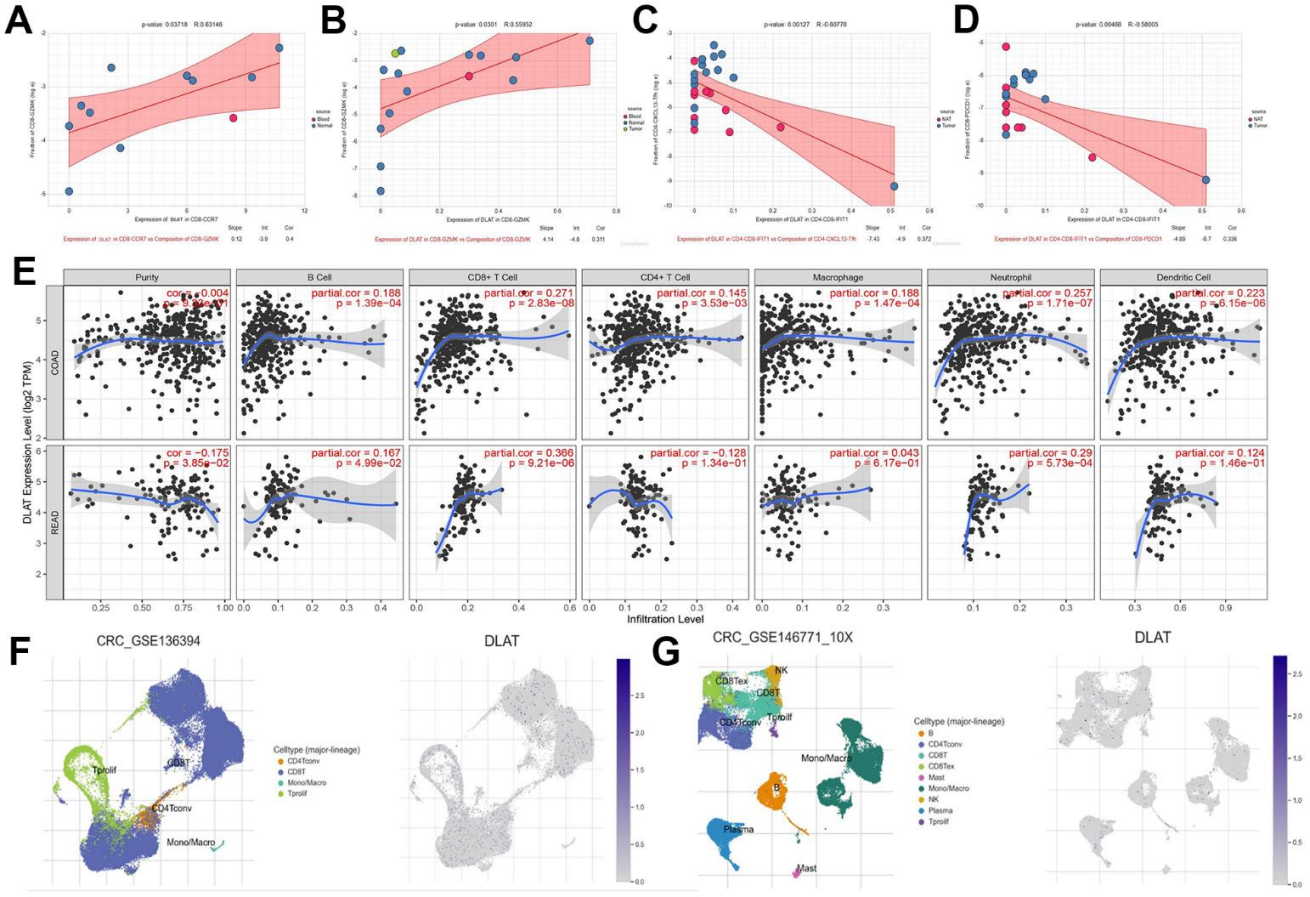
**DLAT shapes the positive tumor microenvironment.** (**A**–**D**) Correlation between expression of DLAT and cell composition (**E**) DLAT expression and immune cell infiltration. (**F**) immune cell landscape and DLAT expression distribution after anti-PD-1treatment (**G**) immune cell landscape and DLAT expression distribution without anti-PD-1 treatment.

Cell communication analysis discovered that CD4-CXCL13-Tfh cells promotes the increase of CD4-CXCL13-Tfh cells (Figure 10D) on the one hand, and leads to the increase of CD4-LEF1-Treg (Figure 10A), CD4-CTLA4-Treg (Figure 10E) and exhausted CD8^+^ T cells CD8-PDCD1 (Figure 10F) moreover by elevating expression of COX17, suggesting that high COX17 expression in CD4-CXCL13-Tfh cells may drive the formation of immunosuppressive TME by inducing T cell exhaustion. Additionally, we also revealed that expression of COX17 in CD4-CCR7-TCF7 and CD4-IL7R-MAL cells were positively correlation with the proportion of CD4-CXCL13-Tfh cells in TME (Figure 11B, 11C). Next, we use TIGER to analyze the CD4-CXCL13-T cell distribution throughout the cancer versus paracancer, and the results showed that tumor tissue contained a proportion of 86.96% of CD4-CXCL13-T cells (Figure 10G, 10H). The expression of COX17 and DLAT in the above cell types was also examined, and the UMAP plot of COX17 expression showed an overlap in the distribution of COX17 and CD4-CXCL13-Tfh cells (Figure 10J), indicating a close association between CD4-CXCL13-Tfh cells and the expression of COX17.

### DLAT shapes the immunoactive tumor microenvironment and enhances anti-PD-1 efficacy

Meanwhile, we also explored the mechanism of DLAT reprogramming TME. High expression of DLAT in CD8-CCR7 and CD8-GZMK T cells infiltration was elevated by CD8-GZMK T cells (p<0.05) (Figure 11A, 11B). CD8-GZMK T cells are commonly denoted as effective T cells, which is the main force in killing tumor cells in anti-tumor immunity. Furthermore, the upregulation of DLAT expression leads to a reduction in the infiltration level of CD4-CXCL13-Tfh and CD8- PDCD1 cells by CD4-CD8-IFIT1 (p<0.01) (Figure 11C, 11D). These outcomes indicated that DLAT has the ability to shape immunoactive TME by elevating the cytotoxic T cells levels and reversing T cells exhaustion. Next, the connection among DLAT and multiple immune cells expression levels through colorectal cancer was analyzed by the TIMER, and the outcomes revealed that the DLAT expression showed a positive association within CD8^+^ T cells, neutrophils, macrophages, B cells, and DC cells infiltration levels (p<0.01) (Figure 11E). Finally, we discovered that DLAT (blue dot) expression was significantly upregulated after anti-PD-1 immunotherapy (Figure 11F, 11G). Taken together, we discovered that DLAT improves the immunotherapy response within CRC individuals by shaping hot TME.

## DISCUSSION

The important role of copper metal in the initiation and proliferation of cancers has made this field of cancer biology a popular focus for investigation. A growing amount of evidence indicates that tumor growth, migration, angiogenesis, and TME formation are significantly influenced by copper ion homeostasis and mitochondrial metabolism [21–23]. Therefore, A RiskScore model was developed utilizing a cuproptosis-related gene signature. The aforementioned model has the ability to precisely forecast the prognosis, clinical characteristics, and response to immunotherapy in CRC patients. Subsequently, we investigated tumor immune microenvironment and pathway activity within the high and low RiskScore groups and screened two hub genes based on this model: COX17 and DLAT. Finally, we combined scRNA-seq analysis to delve into the specific mechanism of TME reprogramming by COX17 and DLAT.

In this study, we firstly revealed alterations in CRGs at both the genetic and transcriptional levels in individuals with CRC. Then taking into account the patient heterogeneity and complexity of the CRC. We constructed the RiskScore model based on 61 CRGs expression profiles utilizing the Lasso-Cox analysis method. Within these, 9 genes connected with the prognosis of colorectal cancer patient. Furthermore, two RiskScore groups demonstrated significant variations in clinical prognosis, immune infiltration, stromal score, TIDE, TMB, etc. Low RiskScore exhibited a more favorable prognosis, more infiltration results of CD8+ T cells, NK cells, and neutrophils, and more sensitivity to immunotherapy such as anti-PD-1 than high RiskScore. This suggests that the high RiskScore and low RiskScore may correspond to the immune-desert phenotype and immune-active phenotype, respectively. The RiskScore model could help deliver ICB therapies more precisely and provide a new idea to break the bottleneck of ICB efficacy. Subsequently, a nomogram was developed by introducing the individual’s age, gender, tumor stage, and RiskScore for enhancing the accuracy of prognostic prediction. Finally, we investigated the pathway variations among the two groups using GSEA and GSVA analysis showed that several pathways critical for the regulation of tumor proliferation, invasion, and immune evasion, including the MAPK pathway, WNT pathway, HYPOXIA pathway, and EGR2 and SOX10 mediated pathways, are upregulated in high RiskScore, and several studies demonstrate the involvement of a hypoxic environment in the formation of immunosuppressive TME [24].

Copper ions are closely related to the immune system and are necessary to maintain a normal immune response [25]. It has also been found that tumors are helped to achieve immune evasion by copper ions mediating the exhaustion of tumor-specific T cells [26, 27]. Our results further elucidated the specific mechanism of this phenomenon, and scRNA-seq analysis found that CD4-CXCL13Tfh induced an increase in CD8-PDCD1 exhausted T cells in CRC TME through elevating expression of COX17, while COX17 also likely induced CD4-CCR7-TCF7 Naïve T cells to become immunosuppressive CD4-CXCL13 T cells and further worsen TME. COX17 is a copper ion chaperone protein that functions by binding intracellular copper and transporting it to specific sites. The role of COX17 in cancer is still unclear. We revealed that COX17 was significantly associated with poor prognostic outcomes within CRC patients and GBM and better prognosis in PCPG. The GSEA analysis found that COX17 mediates tumor proliferation and invasion through the WNT pathway, which is in line with the outcomes of Ramchandani et al., who discovered that by reducing COX17 expression in triple-negative breast malignancy induced copper depletion and thus inhibited tumor metastasis [28]. In contrast, another gene we focused on, DLAT, is likely to activate anti-tumor immunity by inducing an increase in CD8-GZMK T cells. And it reverses the exhaustion of T-cell by reducing CD8-PDCD1 and CD4-CXCL13 T-cell content, forming HOT TME. Previous studies have shown that DLAT functions as the E2 subunit within the PDC complex in the catabolic glucose pathway. It is also associated with diseases such as gastric malignancy, obesity, and non-small cell lung cancer [29, 30]. However, the mechanisms by which DLAT regulates antitumor immunity and reprograms TME have been little studied. Our study revealed a significant connection between DLAT and the prognosis of individuals diagnosed with multiple tumors and may induce the pyroptosis by driving mitochondrial apoptosis, thereby shaping immunoactive TME. In addition to this, DLAT expression levels were increased in CRC after anti-PD-1 treatment, suggesting that it may be a potential prognostic indicator or response biomarker for immune checkpoint blockade therapy.

The results of the cell communication analysis brought CD4-CXCL13 T cells to our attention. According to this study, CD4-CXCL13 T cells clustered in malignant tissues in colorectal cancer and shaped immunosuppressive TME by upregulating COX17, which induced T cell exhaustion and infiltration of Treg. A previous study by Joshua et al. suggested that CD4-CXCL13 T cells in melanoma correlate with survival and macrophage, CD8^+^ T, and B cell activation. This study also found that CD4-CXCL13 T was associated with worse survival in glioblastoma and clear renal carcinoma, etc., which suggesting that there is heterogeneity in the function of CD4-CXCL13T cells [31]. However, the involvement of this cohort of cells in CRC has yet to be investigated, and our study fills the gap to some extent.

Recently, ICB therapy has demonstrated significant potential in treating tumors, but due to the highly immunosuppressive nature of CRC, just a minute proportion of patients experience advantageous outcomes [32]. A prognostic model was developed for predicting the response of individuals suffering from CRC to immunotherapy more accurately and help to implement ICB therapy more precisely. Targeting COX17 and DLAT can also remodel TME and activate anti-tumor immunity, favoring a breakthrough in ICB treatment. However, this study also has some shortcomings. Our research needs prospective CRC cohorts to validate the RiskScore model prognostic efficacy. Additionally, the effect of COX17 and DLAT expression on the cell composition in TME needs to be validated with more investigations.

## CONCLUSIONS

In summary, our research has developed a cuproptosis-related genes-based prognostic model in colorectal cancer that is highly effective in predicting prognosis, TME type, and response to immunotherapy in CRC patients. Moreover, through in-depth analysis of CRGs, we discovered that elevated levels of COX17 expression were observed for the first time in CD4-CXCL13 T cells in CRC mediates T cell exhaustion and Treg infiltration, while DLAT activates anti-tumor immunity by reversing T cell exhaustion and inducing tumor cell pyroptosis. The results validate the clinical significance of CRGs and investigate the process of reprogramming the TME, offering a novel approach for breaking the bottleneck of immunotherapy.

## MATERIALS AND METHODS

### Data gathering and preparation

The bulk RNA sequencing (RNA-seq) data as well as the phenotypic data for the CRC cases was provided by The Cancer Genome Atlas (TCGA, https://portal.gdc.cancer.gov/) database [33]. A cohort under study consisted of 434 samples from patients with colorectal cancer, comprising 383 tumor samples and 51 normal samples. GSE17536, GSE17537, and GSE38832 have been purchased from GEO database, accessible at (https://www.ncbi.nlm.nih.gov/geo/). In a new report by the TSVETKOV team [13] and The Molecular Signature Database (MSigDB) (http://www.gsea-msigdb.org/gsea/msigdb/), we chose 61 cuproptosis-related genes from them for further investigation.

### Construction of a prognosis signature based on 61CRGs

The present study employed the least absolute shrinkage and selection operator (LASSO) method for conducting a regression analysis on 61 genes from the TCGA cohort. Utilizing the “glmnet” R package, the analysis was carried out without overfitting. Eventually, nine genes have been acquired to build the model. RiskScore=∑i=Exp(Xi)×Coef(Xi). Coef (Xi) represents the coefficient of each CRG and Exp(Xi) refers to the expression level of mRNA for each. The TCGA and GEO cohorts have been divided into two distinct groups according to the basis of the optimal risk threshold: a high-risk group (n =172) and a low-risk group (n =200). Lastly, time-related ROC curves were employed to investigate the prognostic precision of the characteristic in forecasting survival outcomes at 1, 3, and 5 years in both high- and low-risk groups.

### Development and verification of nomogram scoring system

Using clinical traits, risk scores, and outcomes from a separate prognostic study, the nomoR software is used to create prediction column line graphs [34]. The final score was calculated utilizing the nomogram scoring system by adding the scores of all variables for each sample. Using the “Timeroc” software program, ROC curve analysis was conducted to evaluate the 1, 3, and 5-year survival rates [35]. The calibration plots of column line plots illustrate the correspondence among the speculated 1, 3, and 5-year survival events and the determined values in terms of expected values.

### Immunoreaction analysis

To compare the variations in TME among two groups categorized as higher and lower risk groups, utilizing the ESTIMATE and CIBERSORT methods, the immune component of CRC was evaluated [36]. The present study assessed the immune checkpoint inhibitor therapy effectiveness within two distinct cohorts utilizing the Tumor Immune Dysfunction and Exclusion (TIDE) computational framework (TIDE, http://tide.dfci.harvard.edu/) relied on their respective dysfunction and exclusion scores [37]. For the purpose of obtaining the IPS, the Cancer Immunome Atlas (https://tcia.at/ was utilized. By conducting a comparative analysis of IPS scores between groups categorized as high-risk and low-risk, it was possible to predict how well CRC patients will respond to different forms of ICI therapy, including PD-1/PD-L1/PD-L2 and CTLA-4 blocking therapy. Utilizing CIBERSORT, EPIC, and MCP-counter algorithms, the immune cells infiltration within the TME was assessed.

### Survival analysis

The Kaplan-Meier (K-M) and log-rank tests were employed throughout the current investigation, utilizing the “survivor” R package, to assess the impact of the RiskScore and nine prognosis-related genes on patient survival.

### Somatic cell mutation analysis

The present study utilized the R package “MAF Tool” to conduct an analysis of DNA mutation data obtained from TCGA. The primary objective was to identify the somatic mutation patterns exhibited by colorectal cancer individuals (CRC) categorized as high-risk group and low-risk group, according to the DNA mutation data. SNV of 61CRGs in the TCGA cohort was also determined by this way.

### Gene set enrichment analysis

The “clusterProfiler” R package was utilized to carry out the pathway enrichment analysis of GO and (KEGG) [38]. According to the data obtained from mRNA expression profiling and MSigDB [39], GSEA [40] was employed to examine the potential signaling pathways that may exist between two distinct groups. With the use of the GSVA package in the R software, the enrichment scores of pathways in each sample were calculated [41].

### Immunohistochemical analysis

The Human Protein Expression Atlas (THPA) is an immunostaining of tissues and cells for differential analysis of protein expression. Presenting proteomic profiles of human tissues based on proteomic, transcriptomic, and systems biology data, combined with tissue microarray-based immunohistochemistry, the database includes individual proteins in all major tissues and organs of the body as well as overall expression [42]. The COX17 and DLAT protein expression levels were evaluated in both normal and tumor tissues through IHC analysis utilizing THPA.

### Single cell RNA sequencing analysis

The scRNA-seq data pertaining to colorectal cancer, specifically GSE139555, GSE136394, and GSE146771, were purchased from the GEO database. We used R package ‘Seurat’ (3.1.1) to process these data. In this study, the Tumor Immune Single-Cell Hub (TISH) database (http://tisch.comp-genomics.org/home/) was utilized to perform gene expression profiles comparative analysis at the single-cell level between groups receiving immunotherapy and those not receiving immunotherapy. The investigation of cellular communication was conducted through the utilization of the software tool ‘cellchat’ and the scTIMER portal. The Tumor Immunotherapy Gene Expression Resource was utilized to analyze gene expression and differentially expressed genes at the level of single cells (TIGER, http://tiger.canceromics.org/).

### Cell culture and quantitative real-time polymerase chain reaction (RT-qPCR)

The colorectal cancer cell line HCT116 and normal colon epithelial cell line NCM-460 were purchased from Fuheng Biotechnology (Shanghai, China). These cells were cultured in Dulbecco’s Modified Eagle Medium (DMEM) (Gibco, USA) with 10% fetal bovine serum (FBS) (Gibco, USA) and 1% penicillin-streptomycin in an atmosphere containing 5% CO2 at 37° C.

For RT-qPCR assay, we first extracted total RNAs from cells using AG RNAex Pro reagent (Accurate Biology, AG21102). After then, these RNAs were reverse transcribed into cDNA using Evo M-MLV Kit (Accurate Biology, AG11705). The cDNA was eventually used for RT-qPCR analysis using SYBR Green Pro Taq HS Premix (Accurate Biology, AG11701). All the reactions were performed in StepOnePlus™ instrument. The 2^-ΔΔCt^ strategy was applied to compute the relative mRNA level of genes. Human GAPDH was utilized to normalize expression levels. The sequences of the primers were as follows:

Human GAPDH: 5’- GGAGCGAGATCCCTCCAAAAT GGCTGTTGTCATACTTCTCATGG-3’

COX17: 5’- GGTCGGGTCTCTGTTGACTT TTGCCGTTCTCCTCTCTCTC-3’

DLAT: 5’- CGGAACTCCACGAGTGACC CCCCGCCATACCCTGTAGT-3’

### Statistical analysis

The R version 4.0.4 was utilized to perform the statistical analyses. Pearson or Spearman correlation analysis were employed throughout this investigation to evaluate the correlation between two distinct groups. In order to generate survival curves, the KM approach was applied, and were evaluated utilizing the log-rank test. The Lasso regression analysis was conducted in the investigation to evaluate the prognostic significance of the variables on the hazard. The Students t-test statistical method was employed to evaluate the variations within the groups. p < 0.05 was reported as statistically significant difference.

### Data availability statement

All datasets and codes used during the current study are available from the corresponding author upon reasonable request.

## Supplementary Material

Supplementary Figures

Supplementary Table 1

Supplementary Table 2

Supplementary Table 3

Supplementary Table 4
