# Supplementary material for: Integrative analysis of single-cell and bulk RNA seq to reveal the prognostic model and tumor microenvironment remodeling mechanisms of cuproptosis-related genes in colorectal cancer
Source: Aging (Albany NY). 2023 Dec 8;15(23):14422–44. doi: 10.18632/aging.205324 (PMC10756095; doi:10.18632/aging.205324)
Supplement: Supplementary Figures [file aging-15-205324-s001.pdf]

SUPPLEMENTARY FIGURES

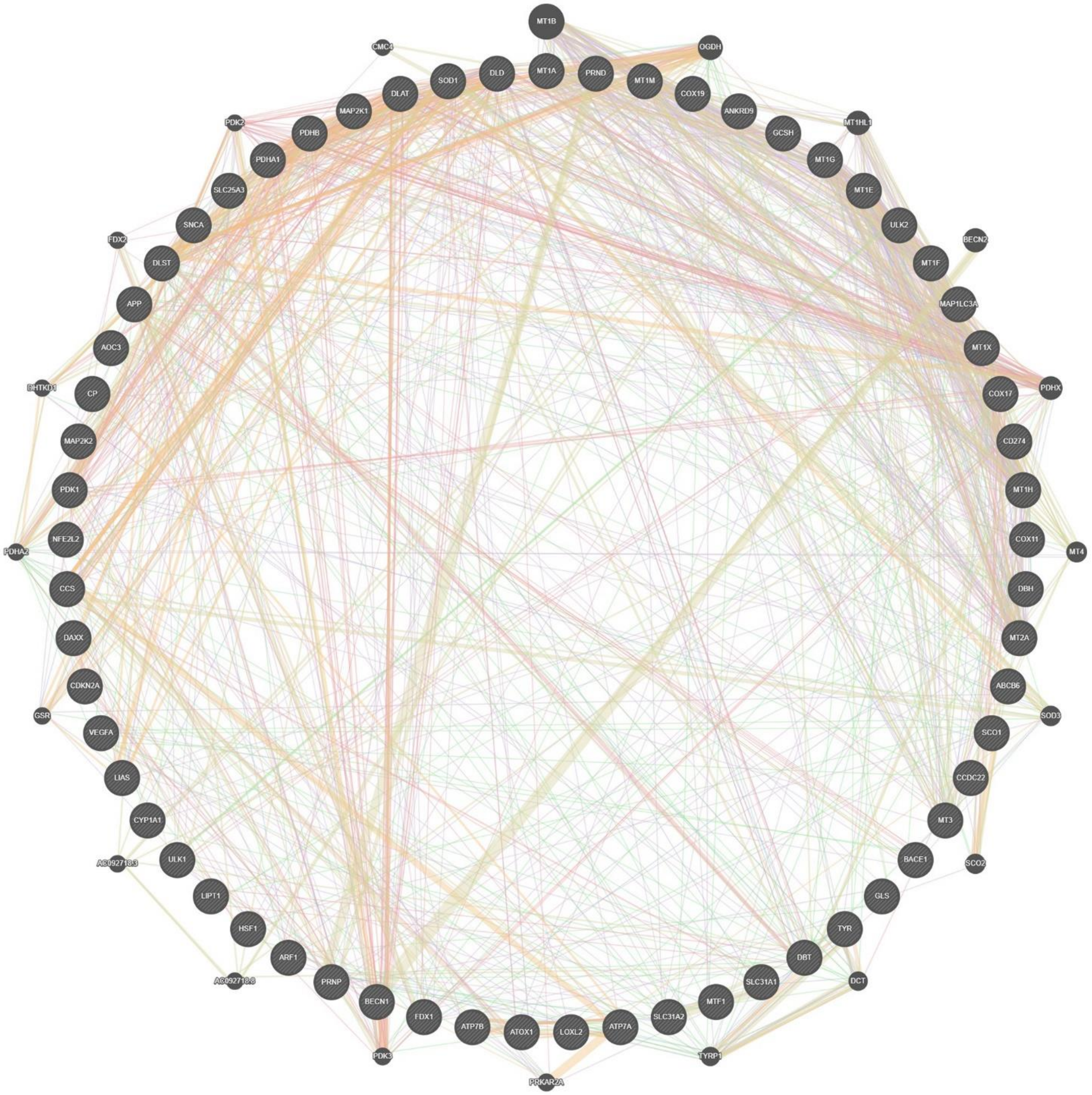

Supplementary Figure 1. PPI plot of 61 cuproptosis-related gene by GeneMANIA.

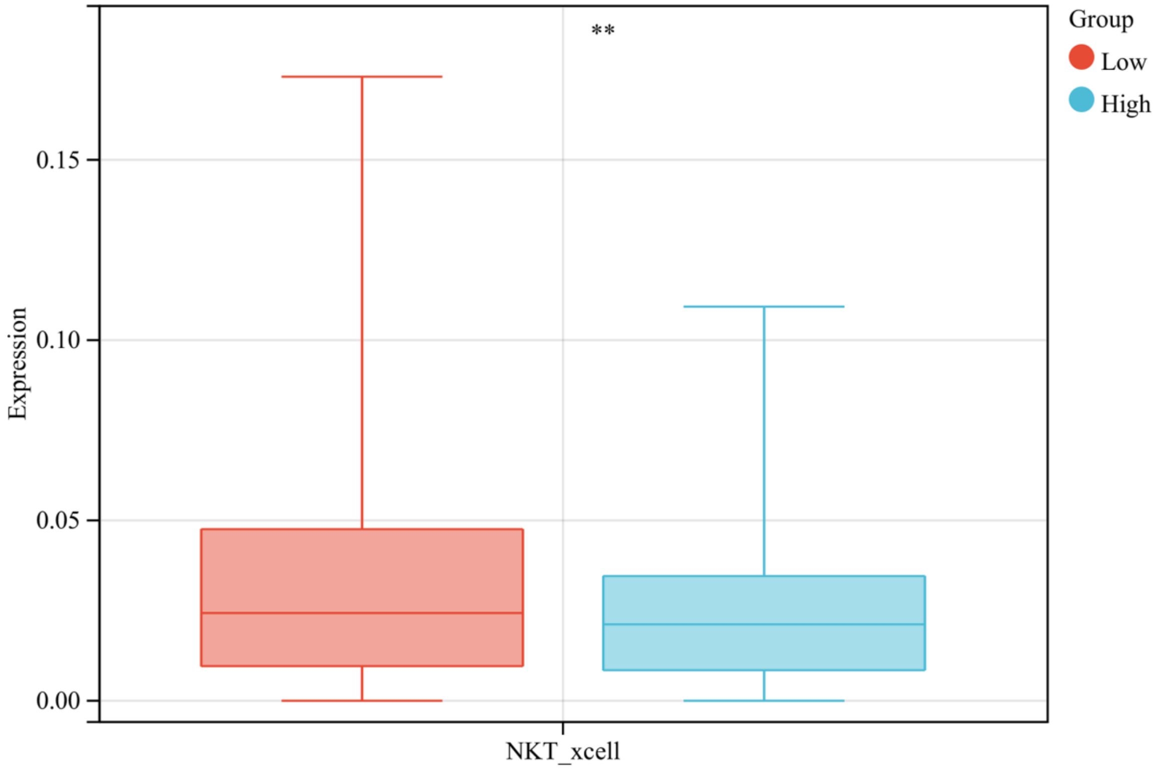

Supplementary Figure 2. The infiltration level of NKT cells in low- and high-risk groups.

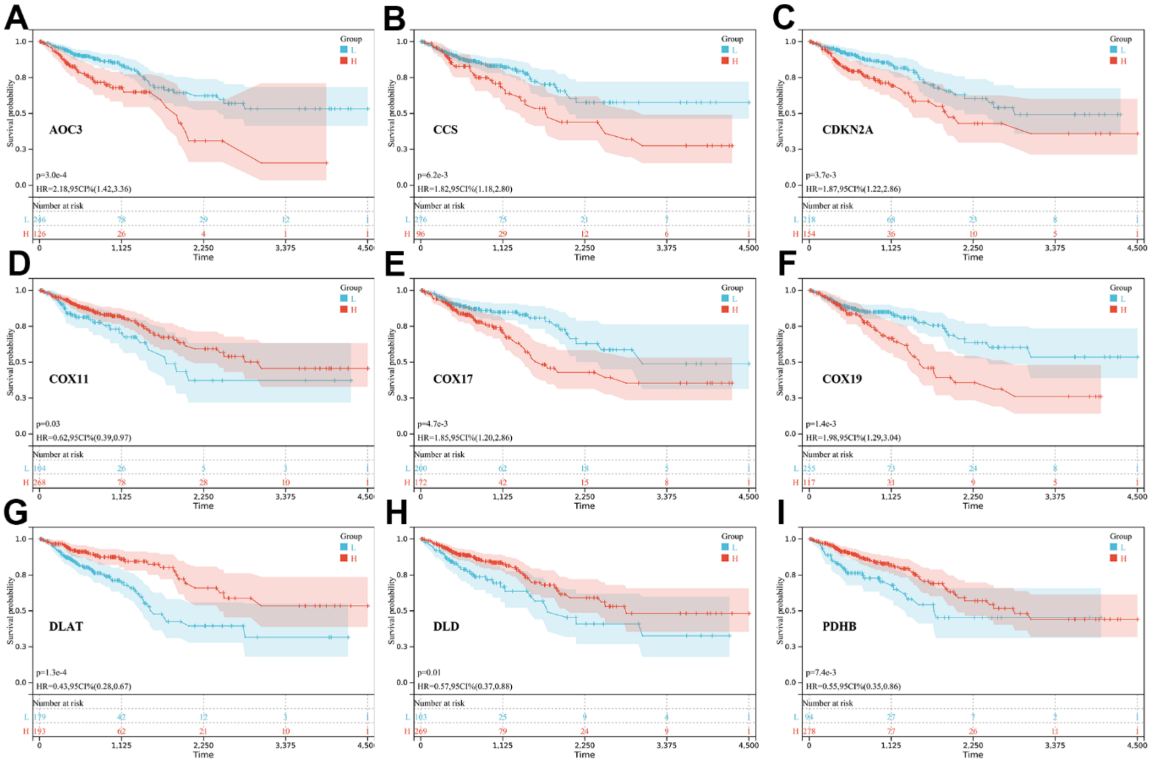

Supplementary Figure 3. KM plot of 9 prognosis-related CRGs. The KM plot of AOC3 (**A**), CCS (**B**), CDKN2A (**C**), COX11 (**D**), COX17 (**E**), COX19 (**F**), DLAT (**G**), DLD (**H**), PDHB (**I**).

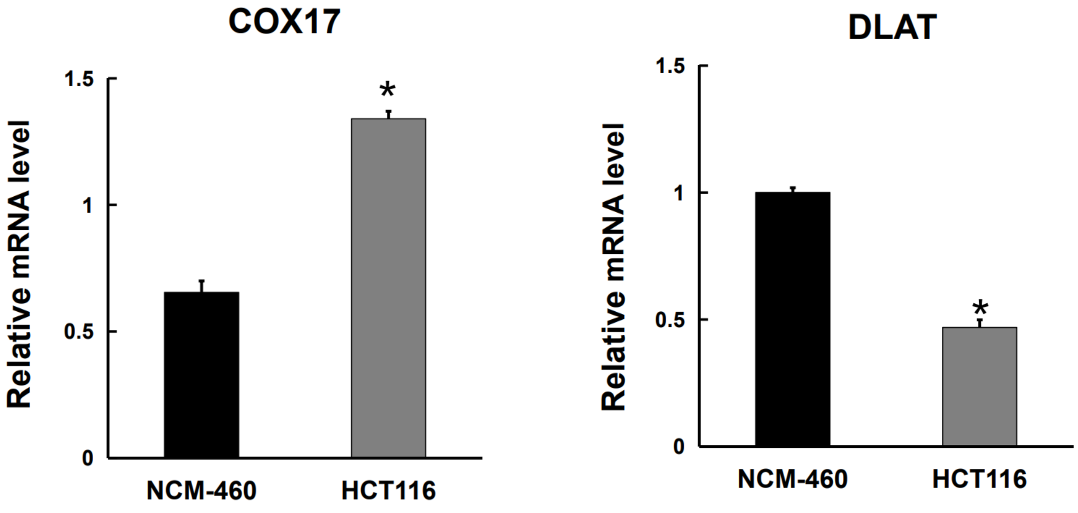

Supplementary Figure 4. Relative mRNA level of COX17 and DLAT in HCT116 and NCM-460 cells.

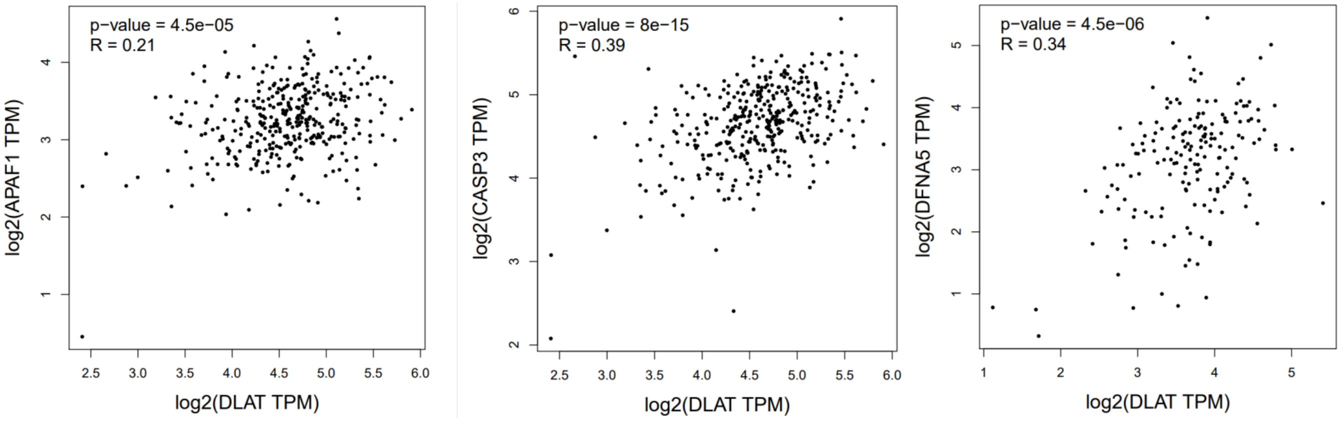

Supplementary Figure 5. Correlation analysis of DLAT with APAF1, CASP3 and GSDME (also known as DFNA5).

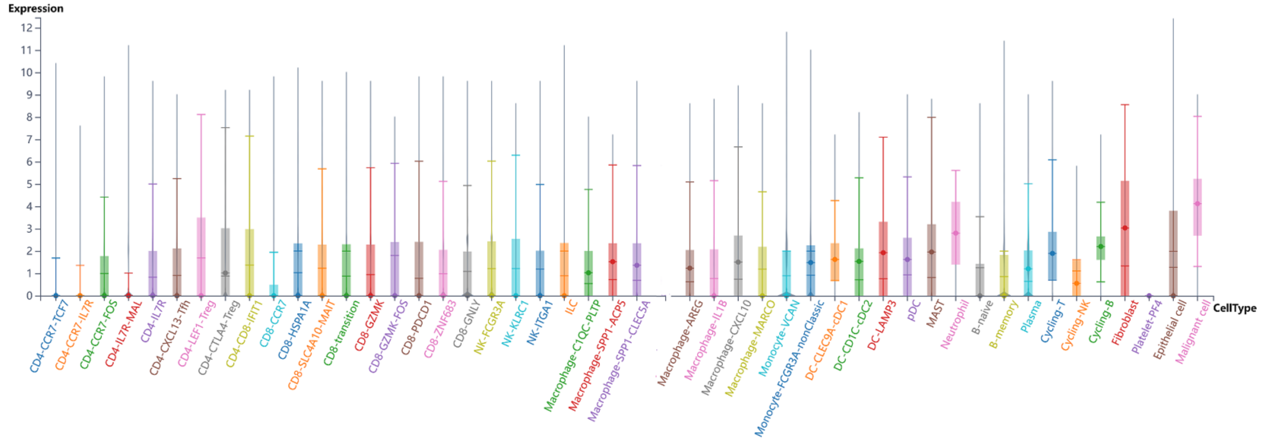

Supplementary Figure 6. The expression of COX17 in different cells.

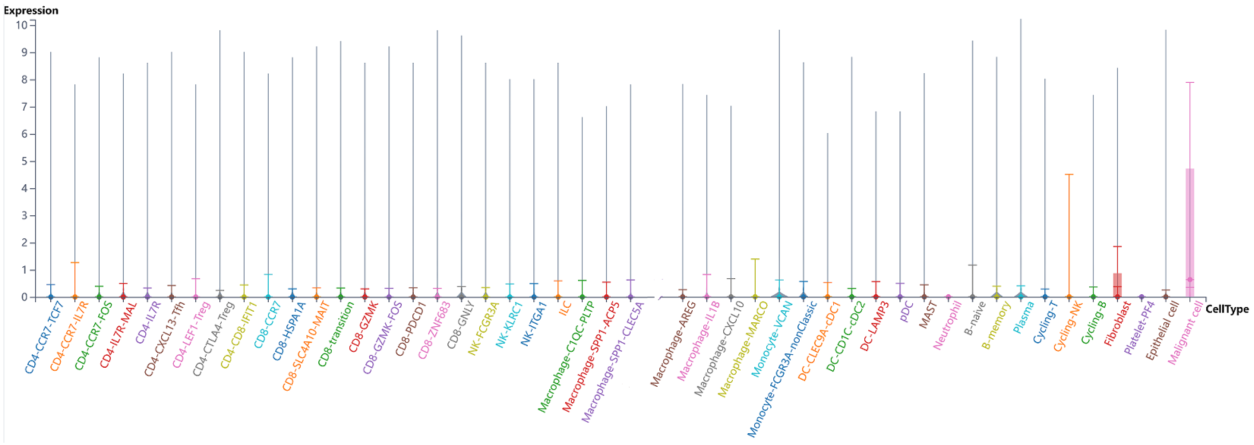

Supplementary Figure 7. The expression of DLAT in different cells.
