# Supplementary material for: Integrative analysis of single-cell and bulk RNA seq to reveal the prognostic model and tumor microenvironment remodeling mechanisms of cuproptosis-related genes in colorectal cancer
Source: Aging (Albany NY). 2023 Dec 8;15(23):14422–44. doi: 10.18632/aging.205324 (PMC10756095; doi:10.18632/aging.205324)
Supplement: Supplementary Table 4 [file aging-15-205324-s005.pdf]

Supplementary Table 4. The 9 signature genes and their coefficients.

Gene	Coefficients
COX19	0.0723218467945424
COX11	-0.0159333955707089
COX17	0.313420628697771
AOC3	0.0624032376941578
CCS	0.0497832615996111
DLAT	-0.242743143130727
PDHB	-0.129752029159547
DLD	-0.0863956442851258
CDKN2A	0.054461573615976
